# A meta-analysis and systematic review of changes in joint position sense and static standing balance in patients with whiplash-associated disorder

**DOI:** 10.1371/journal.pone.0249659

**Published:** 2021-04-08

**Authors:** Masood Mazaheri, Deepa Abichandani, Idsart Kingma, Julia Treleaven, Deborah Falla

**Affiliations:** 1 Centre of Precision Rehabilitation for Spinal Pain (CPR Spine), School of Sport, Exercise and Rehabilitation Sciences, University of Birmingham, Birmingham, United Kingdom; 2 Lecturer in Physiotherapy, Institute of Health and Social Care, London South Bank University, London, United Kingdom; 3 Department of Human Movement Sciences, Faculty of Behavioural and Movement Sciences, Vrije Universiteit Amsterdam, Amsterdam Movement Sciences, Amsterdam, The Netherlands; 4 Division of Physiotherapy, School of Health and Rehabilitation Sciences, The University of Queensland, Brisbane, Australia; Universiteit Antwerpen, BELGIUM

## Abstract

**Objective:**

To synthesise and analyse the current evidence regarding changes in joint position sense (JPS) and standing balance in people with whiplash-associated disorder (WAD) taking the presence or absence of dizziness into account.

**Data sources:**

PubMed, CINAHL Plus, Web of Science, Embase, MEDLINE and APA PsycINFO were searched by two independent reviewers from inception until August 2020 and reference lists of all included studies were also reviewed.

**Study selection:**

Only cross-sectional studies that measured JPS and/or standing balance between people with WAD vs. healthy controls (HC) or people with WAD complaining of dizziness (WAD_D_) vs. those not complaining of dizziness (WAD_ND_) were selected.

**Data extraction:**

Relevant data were extracted using specific checklists and quality assessment was performed using Downs and Black Scale (modified version).

**Data synthesis:**

Twenty-six studies were included. For JPS, data were synthesized for absolute error in the primary plane of movement for separate movement directions. For standing balance, data were synthesized for traditional time- and frequency domain sway parameters considering the conditions of eyes open (EO) and eyes closed (EC) separately. For meta-analysis, reduced JPS was observed in people with WAD compared to HC when the head was repositioned to a neutral head position (NHP) from rotation (standardised mean difference [SMD] = 0.43 [95%: 0.24–0.62]) and extension (0.33 [95%CI: 0.08–0.58]) or when the head was moved toward 50° rotation from a NHP (0.50 [0.05–0.96]). Similarly, people with WAD_D_ had reduced JPS compared to people with WAD_ND_ when the head was repositioned to a NHP from rotation (0.52 [0.22–0.82]). Larger sway velocity and amplitude was found in people with WAD compared to HC for both EO (0.62 [0.37–0.88] and 0.78 [0.56–0.99], respectively) and EC (0.69 [0.46–0.91] and 0.80 [0.58–1.02]) conditions.

**Conclusion:**

The observed changes of JPS and standing balance confirms deficits in sensorimotor control in people with WAD and especially in those with dizziness.

## Introduction

‘Whiplash associated disorder’ (WAD) is a term to describe symptoms associated with a whiplash injury [1], caused by a sudden acceleration-deceleration movement of the neck, most commonly following a motor vehicle accident. The acute symptoms include neck pain as well as dizziness and pain in other body regions. Symptoms of WAD persist even one year after injury in ~50% of people [2], indicating delayed recovery in a significant proportion of patients.

After pain, dizziness is one of the most common complaints in persistent WAD; a symptom likely due to altered cervical afferent input to the sensorimotor control system [3]. People with WAD may demonstrate alterations in joint position sense (JPS) and standing balance [3], two domains of sensorimotor control. Plausible causes of sensorimotor dysfunction include damage to mechanoreceptors due to trauma [4], morphological changes of neck muscles [5], pain and inflammation [6] and activation of sympathetic nervous system as a consequence of high levels of stress [7]. Nevertheless, there is conflicting evidence with some studies reporting impaired JPS and balance [8, 9] in people with WAD whereas other studies report no change [10, 11]. Thus, there is a need for a systematic review to determine whether these aspects of sensorimotor control are impaired in people with WAD.

Thus far, changes in JPS in people with WAD have been considered in one systematic review by de Vries et al. [12] and balance in two systematic reviews by Silva et al. [13] and Ruhe et al. [14]. However, these reviews were limited to qualitative analysis of the findings [12–14], and in one case [12] a substantial number of relevant studies, were not included. These limitations, together with the need to consider newly published research since the last literature search performed for JPS (December 2014 [12]) and for balance (November 2010 [13] and January 2011 [14]), justified the need to conduct the current systematic review that uses a quantitative approach to synthesize the available evidence. The present study specifically investigated the role of WAD and dizziness on the afore-mentioned outcomes by comparing people with WAD and healthy controls (HC) and people with WAD presenting with dizziness (WAD_D_) versus those not presenting with dizziness (WAD_ND_) due to potential greater deficits in sensorimotor control in those complaining of dizziness.

## Materials and methods

### Eligibility criteria

The PICOS (P: Patient/population; I: Intervention; C: Comparison; O: Outcome; S: Study design) framework, for which we defined P as ‘WAD’ or ‘WAD_D_’, C as ‘healthy controls’ or ‘WAD_ND_’, O as ‘JPS’ or ‘balance’ and S as ‘cross-sectional study design’, was utilized to inform the eligibility criteria. The I component was not applicable.

#### Population

Only studies that measured either ‘JPS’, ‘standing balance’ or both in human participants with WAD classified as Grade I, II or III were included. Comparison had to be between people with WAD and HC or between WAD_D_ and WAD_ND_ groups.

#### Outcome

For JPS, studies were included if they used target matching tasks, i.e. repositioning the head to a neutral or target position, in which the target is achieved by moving the head and neck on the stationary trunk. Studies were excluded if they used trajectory registration tasks that required the participant to follow a visual target, such as Figure of 8 [15, 16], The Fly [17, 18], or Zigzag [16] tests, as they measure movement sense and not position sense. For standing balance, studies were eligible if they measured postural sway derived from centre of pressure (COP) recordings during bipedal static standing on a force platform. Studies that applied external perturbation using for instance platform translations or rotations were excluded. In case of duplicate publication or publications with similar data, the original version of the article was included in this review.

#### Study design

Only observational cross-sectional studies, published in English, were included in this review.

### Information sources and search strategy

The literature was searched in several electronic databases including PubMed, CINAHL Plus (EBSCOhost), Web of Science, Embase (OVID), MEDLINE (OVID) and APA PsycINFO (OVID) from their inception until August 2020. The search of keywords, across title, abstract and subject headings, and their database-specific variants resulted in a specific search string which can be found in Table 1. In addition to the electronic search, reference lists of selected articles from the original databases were hand searched for additional publications.

**Table 1:** Search strategy used in electronic databases including PubMed, CINAHL Plus (EBSCOhost), Web of Science, Embase (OVID), MEDLINE (OVID) and APA PsycINFO (OVID).

Keyword	Search terms
Whiplash	Whiplash OR Whiplash injur* OR Neck injur* OR Cervical injur* OR Neck pain OR Cervical pain OR Neck lesion OR Cervical lesion OR Neck trauma OR Cervical trauma OR Neck dysfunction OR Cervical dysfunction
Joint position sense	Propriocept* OR Kinesthe* OR Mechanorecept* OR Muscle spindle OR Motion threshold OR Movement threshold OR Reposition* OR Position sense OR Movement sense OR Movement detection OR Motion perception
Standing balance	Postural balance OR Postural control OR Sway OR Center of pressure OR Centre of pressure OR COP OR Posturogra* OR Stabilogra* OR Force plate OR Force platform OR Postural stabil* OR Body stabil* OR Postural equilibrium OR Postural function OR Postural behaviour OR Postural behavior OR Postural performance OR Postural regulation OR Postural strategy OR Postural dysfunction OR Body balance OR Body equilibrium
The final search string was constructed using (Whiplash) *AND* ((Joint position sense) *OR* (Standing balance))	

### Study selection

Two reviewers (MM, DA) independently screened the relevant citations against the pre-determined eligibility criteria. A third reviewer (DF) was consulted in the event of disagreement throughout the stages of the review. Authors were contacted if full texts were unavailable or, if further clarification was required on the study population or outcome.

### Data extraction

Two reviewers (MM, DA) extracted data related to demographic and clinical characteristics of patient populations including inclusion/exclusion criteria, sample size, age, height, weight, body mass index, variables matched (or statistically adjusted), WAD grade, time since injury or initiation of symptoms, intensity of pain and pain-related disability. To describe the protocol of the included studies measuring JPS the following data were extracted: measurement method, equipment, start position, movement performed, movement velocity, target position, number of practice and measurement trials and outcome measures. For standing balance, the extracted relevant data included: equipment, test condition, instruction, foot and hand position, duration and number of measurement trials, sampling frequency and outcome measures. Mean and SD or standard error of mean (SEM) of main outcomes were also extracted.

### Methodological quality

A modified version of Downs and Black Scale [19], designed for assessing the quality of both randomized and non-randomized studies, was used for quality rating in the present study. The modified version consists of 4 domains (11 items) including quality of reporting (6 items), the generalizability or external validity (1 item), internal validity (3 items) and the adequacy of sample size or study power (1 item) (Table 2). Two authors (MM, DA) separately assessed the quality of the eligible studies.

**Table 2:** Quality rating instrument adjusted specifically for the current review (informed mainly by Downs and Black Scale).

Item	Scoring guideline
*Reporting*	
1. Is the hypothesis/aim/objective of the study clearly described?	
2. Are the inclusion/exclusion of the participants included in the study clearly described?	
3. Are the demographic characteristics of the participants included in the study clearly described?	The question is answered ‘yes’ if information about *age and gender* of people with WAD is provided.
4. Are the clinical characteristics of the participants included in the study clearly described?	The question is answered ‘yes’ if information about *injury grade* and *time since injury/initiation of symptoms (such as pain)* and *pain* or *disability level* in people with WAD is provided.
5. Is the treatment history of the WAD participants clearly described?	
6. Does the study provide estimates of the random variability in the data for the main outcomes?	The question is answered ‘yes’ if studies have provided quantitative values of the mean and the standard error or standard deviation.
*External validity*	
7. Were the participants that were asked to participate in the study representative of the entire population from which they were recruited?	The question is answered ‘yes’ if the studies have used several recruitment methods (e.g. self-report, hospital, insurance companies, etc.) [20].
*Internal validity/bias & confounding*	
8. Was an attempt made to blind those measuring the main outcomes?	
9. Were the main outcome measures, i.e. proprioception and standing balance, reliable?	Joint position sense The question is answered ‘yes’ if JPS was measured at least 6 times [21]. Standing balance The question is answered ‘yes’ if bipedal standing balance was measured at least 3 times and/or each trial lasted not less than 90 sec. [22].
10. Were controls matched with WAD participants in important characteristics?	The question is answered ‘yes’ if appropriate matching on confounders, i.e. age and gender, was performed or if adjustment for these variables is made in the statistical analysis.
*Power*	
11. Was there an appropriate sample size of WAD participants and controls?	The question is answered ‘yes’ if a sample size justification or power description provided.

### Data analysis

For JPS, data were pooled when two or more studies were similar with respect to all of the following aspects: JPS task, type of movement (pure flexion, extension, rotation and side-bending; no complex multi-planar movement) and type of error used for the outcome. For standing balance, when a variety of time- and frequency-domain COP measures were provided in a study, only the traditional time-domain measures (i.e. sway velocity and sway amplitude) were considered for analysis. We reported frequency-domain measures if they were the only outcome measure in the included study. COP data were synthesized in eyes open (EO) and eyes closed (EC) conditions separately.

For meta-analysis, mean, standard deviation (SD) of JPS error and COP parameters and number of subjects were required to be extracted from single groups. To combine mean, SD and number of subjects of two or more subgroups into a single group number, we used the formula recommended by the Cochrane handbook for systematic reviews (https://training.cochrane.org/handbook/current). Where SEM was only reported, SD was calculated. When numerical data was only presented in figures, we used WebPlotDigitizer software (https://automeris.io/WebPlotDigitizer) to extract the mean and SD or SEM from figure images.

The above variables were entered into Review Manager 5.3 software with the following input parameters: continuous data (data type), inverse variance (statistical method), random effects (analysis model), standardized mean difference (SMD; effect measure), totals and sub-totals (totals) and 95% (study confidence interval [CI] and total CI). SMD, CI, *p*-value and *I*^2^ index were calculated and demonstrated in the forest plot. The effect size represented by SMD was interpreted according to Cohen’s suggestion as small, SMD = 0.2; medium, SMD = 0.5; and large, SMD = 0.8 [23]. *p*-value smaller than 0.05 was considered as significant. The threshold to interpret *I*^2^, representing the amount of statistical heterogeneity, includes low, *I*^2^ = 25%; moderate, *I*^2^ = 50%; and high, *I*^2^ = 75% [24]. When meta-analysis was not possible, no overall effect was presented in the forest plot. As methodological quality might explain possible heterogeneity among studies, the total quality score was compared using Mann-Whitney *U* test between studies finding differences in JPS and standing balance between people with WAD and HC compared to those that did not.

## Results

### Literature search

**Fig 1:**
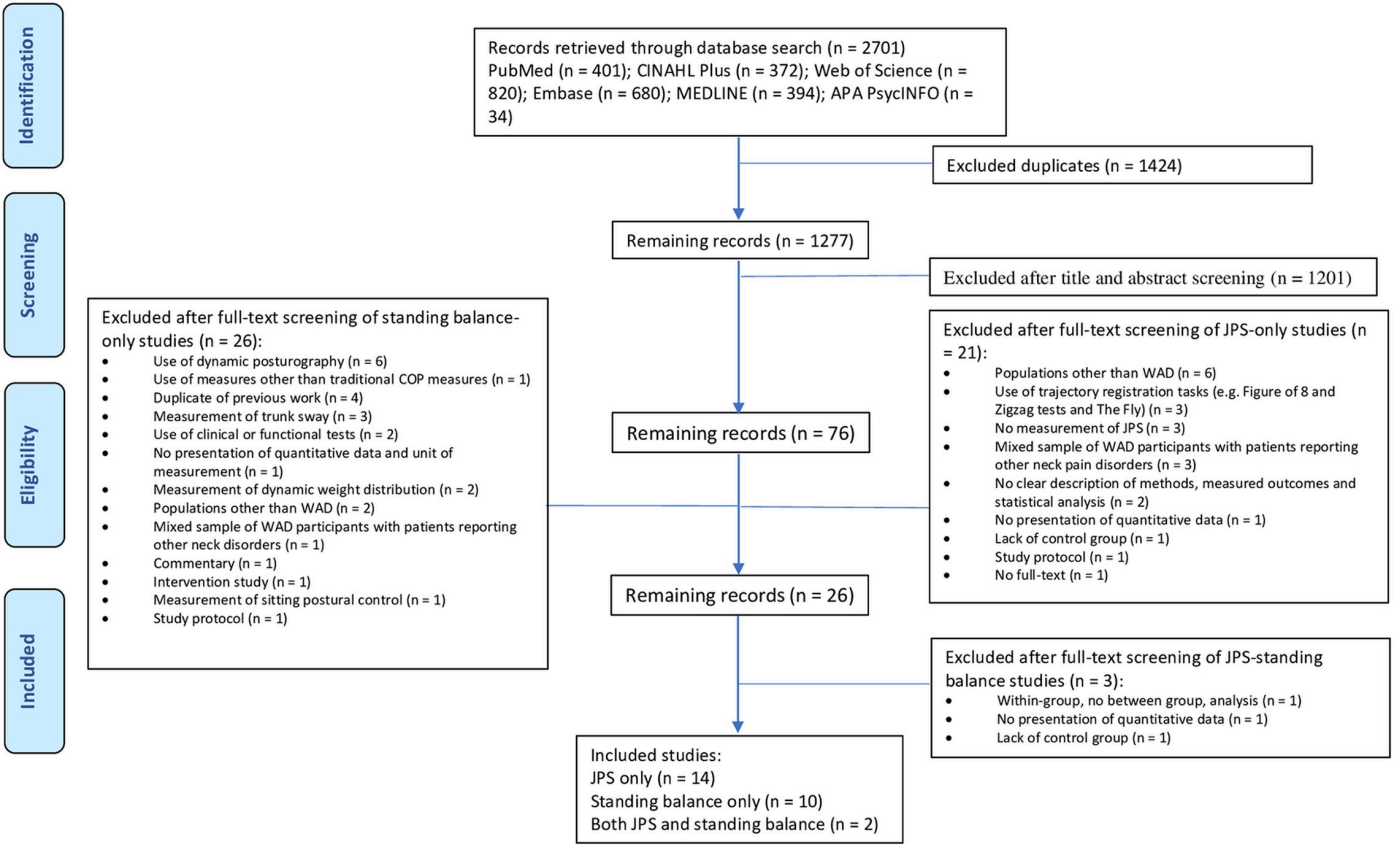
Flowchart of study selection.

In total, 2701 potentially relevant studies were identified after the original search of databases. Once duplicates were removed and title, abstract and full-texts were screened, 26 studies remained, from which 16 studies were included for review of JPS [8, 10, 15, 25–37] and 12 studies for review of standing balance [9, 11, 25, 36, 38–45], with two studies reporting both JPS and standing balance [25, 36] (see Fig 1 showing flowchart of study selection).

### Joint position sense

#### Description of the participants

All of the 16 included studies assessed the difference between people with WAD and HC, among which only 2 studies assessed the difference between WAD_D_ and WAD_ND_ groups [29, 35]. The studies investigating the difference between people with WAD and HC recruited a total of 1068 participants to compare the difference between WAD (N = 601; range: 7–102) and HC (N = 467; range: 11–57) (Table 3). With the exception of one study that measured JPS within 1 month post-injury [34], the majority of studies assessed participants with chronic pain (>3 months). Pain intensity experienced by the WAD group was reported only in 7 studies [10, 15, 25, 26, 33, 35, 37], using 0-10-point pain rating scales, including visual analogue scale (VAS) and numerical rating scale (NRS). Excluding one study that measured pain during the last week [33], other studies reported current pain, with an average of 5.0 (range over studies: 3.4–6.6). Pain-related disability was reported in 8 studies; of the studies using the Neck Disability Index (NDI) [10, 31–34], the average score was 36.5% indicating moderate disability.

**Table 3:** Inclusion/Exclusion criteria and demographic characteristics of all included studies.

First author (year)	Inclusion/exclusion criteria	WAD (N; age; sex [f/m]; height; weight; BMI)	Controls (N; age; sex [f/m]; height; weight; BMI)	Variables matched or statistically adjusted	WAD grade	Time since injury or initiation of symptoms	Pain/Disability/Fear of movement
Armstrong (2005) [10]	Grade II or III WAD At least 1 whiplash injury > 3 mon, < 5 years No intervention at the time of study No previous history of head injury, spinal fracture/dislocation/surgery, systemic inflammatory disorders, neurological disorders, Meniere’s disease, vertigo, medication for vertigo, inner ear damage and large metallic implants	23; 41.2 (11.9); 15/8; NR; NR; 24.7 (4.7)	23; 33.9 (12.1); 13/10; NR; NR; 23.4 (3.2)	Age, sex and BMI	20 Grade II; 3 Grade III	Injury > 3 mon, < 5 years Symptoms: 28.5 (19.5) mon	Pain Scale (0–10): 5.52 (1.86) NDI (0–100%): 24 (11)
Bianco (2014) [38]	Grade I or II WAD No grade III or IV WAD, orthopaedic and nervous pathology, use of drugs that could affect nervous system, head disorders that could affect balance, obesity (BMI > 30), serious visual and vestibulo-cochlear dysfunction	20; 30.35 (8.10); 0/20; 170.90 (8.62); 68.00 (14.15); 23.23 (4.48)	22; 32.09 (8.94); 0/22; 168.55 (6.69); 67.41 (10.31); 23.66 (2.90)	Age, sex, height, weight and BMI	Grade I or II	Symptoms: 3–12 mon	NR
De Pauw (2018) [25]	Grade II A, B or C WAD Persistent neck pain > 3 mon, Verbal NRS > 3/10, NDI ≥ 10/50 Stable medication intake > 1 mon prior to study participation No major depression or psychiatric illness, neurologic, metabolic, cardiovascular disorders, inflammatory conditions, fibromyalgia, chronic fatigue syndrome, neck or should surgery, pregnancy, 1 year postnatal, intake of non-opioid analgesics 48 hours before participation, heavy physical exertion and consumption of alcohol, caffeine and nicotine on the day of testing	35; 47.00 (1.11); 35/0; NR; NR; 22.30 (3.64)	30; 30.45 (1.15); 30/0; NR; NR; 21.83 (3.81)	Age (adjusted), sex and BMI	35 Grade II	Symptoms (pain)> 3 mon; 86.62 (86.66) mon	Verbal NRS (0–10), current pain: 5.00 (2.70)
Endo (2008) [39]	Grade II WAD Neck pain and vertigo or dizziness > 6 mon No Grade I, III or IV, head injuries, fracture, or dislocation of the cervical spine, lost consciousness, previous history of neck injury or vertigo and dizziness before the motor accident	32; 39.0 (10.1); 19/13; NR; NR; NR	20; 37.9 (9.3); 4/16; NR; NR; NR	NR	Grade II	Symptoms (neck pain, vertigo or dizziness) > 6 mon	NR
Eriksson (2019) [40]	Grade II or III	54; 37; 40/14; NR; NR; NR	30; 26; 17/13; NR; NR; NR	NR	Grade II or III	Symptoms > 12 mon	NR
Feipel (2006) [8]	Grade I, II or III WAD Age > 20 No history of head and neck surgery, known spine pathologies	29; 37 (14); 18/11; 169 (10); 66 (13); NR	26; 35 (11); 14/12; 169 (7); 63 (10); NR	Age, sex, height and weight	8 Grade I/II; 21 Grade III	Injury: < 6 mon (29% subjects) and > 2 years (in 39% subjects); 31 (32) mon	NR
Field (2008) [9]	Grade II WAD Pain duration > 3 mon Age 18–45 years NDI > 10/100% No dizziness or unsteadiness, loss of consciousness at the time of injury, current or past lower limb problems, known vestibular pathology, significant visual or hearing deficits, neurological deficits, Type II diabetes, abnormal blood pressure, or diagnosed psychiatric disorders Not taking medication such as antipsychotic and narcotic medication and not consuming alcohol for 24 h prior to testing	30; Mean (SE), 30.3 (1.3); 24/6; NR; NR; NR	30; Mean (SE), 26.8 (1.3); 23/7; NR; NR; NR	Age and gender	Grade II	Symptoms (pain) > 3 mon	VAS (0–10), Mean (SE), current resting pain: 3.2 (0.4) NDI (0–100%), Mean (SE): 36.9 (2.8)
Grip (2007) [26]	WAD Grade I or II Symptoms longer than 3 mon	22; 49 (15); 17/5; NR; NR; NR	24; 50 (18); 16/8; NR; NR; NR	NR	WAD Grade I or II	Symptoms > 3 mon	VAS (0–10), current pain: 6.6 (1.9) Neck Pain and Disability Scale (0–100): 59.8 (17.0) Disability Rating Index (0–100): 50.5 (20.3) Fear Avoidance Beliefs Questionnaire Physical activity subscale: 13.1 (5.5) Work subscale: 23.2 (9.2)
Heikkilä (1996) [27]	Time since car accident: 2–3 yrs (range: 6 mon-10 yrs) Pain and impaired mobility after accident with persistent neck pain Suffered quality of life and vocational satisfaction Limited activities of daily living and stress tolerance	14; 36 (23–47); 7/7; NR; NR; NR	34; 35 (26–53); 21/11	NR	NR	Injury > 6 mon, < 10 years; 24–36 mon	NR
Heikkilä (1998) [28]	WAD Grade II or III No history of head injury, unconsciousness, neck fracture or dislocation, neck injury or pain	27; 38.8 (18–66); 13/14; NR; NR; NR	39; 35 (26–53); 24/15; NR; NR; NR	NR	WAD Grade II or III	Injury > 15 mon, < 26; 12–24 mon	NR
Hill (2009) [29]	Neck rotation > 30° No unconsciousness or concurrent head injury associated with WAD or a previous history of dizziness No medication > 24 h before study	WAD _D_ 50; 35.5 (8.1); 38/12; NR; NR; NR WAD _ND_ 50; 35.0 (9.5); 38/12; NR; NR; NR	50; 29.5 (8.3); NR; NR; NR; NR	Age (WAD _D_ & WAD _ND_)	NR	Injury > 3 mon; WAD _D_: 16.8 (4.2–36) mon; WAD _ND_: 19.2 (3.6–36) mon	NR
Juul-Kristensen (2013) [41]	Females aged 18–60 Chronic neck pain > 2 yrs since whiplash trauma, NDI > 10/50 No brachial neuropathy, intrusive illnesses, such as cardiovascular disease, life-threatening and neurological diseases; pregnancy; injury/pain in the hip, knee or ankle, that could possibly influence postural control Not being in progressive physical or medical treatment; being in an unstable social or work situation; or waiting for the results of unresolved insurance claim	10; 37.7 (13.64); 10/0; NR; 72.92 (22.22); 25.36 (8.86)	10; 35.9 (12.5); 10/0; NR; 63.88 (10.06); 22.88 (3.17)	Age, sex, weight and BMI	NR	Symptoms > 2 years	NRS (0–10): 4.73 (1.99) NDI (0–100%): 41.20 (14.42)
Kristjansson (2003) [15]	Symptoms between 3–48 mon No previous history of neck pain, disease affecting neck or throat, rheumatic or neurologic diseases	22; 33.4 (10.6); 11/11; NR; NR; NR	21; 26.9 (6.4); 11/10; NR; NR; NR	Age and sex	NR	Symptoms (pain) > 3, < 48 mon; 21.9 (12.5) mon	VAS (0–10), current pain: 3.37 (2.8) NPQ (0–100%): 39.98 (18.0)
Loudon (1997) [30]	One or more whiplash injuries > 3 mon, < 2 years Complains of pain and limited range of motion	11; 42 (8.7); 9/2; NR; NR; NR	11; 43 (3.1); NR; NR; NR; NR	Age and sex	NR	Injury > 3, < 24 mon	NR
Michaelson (2003) [42]	Neck pain > 6 mon No neurological disease, signs of brain damage, rheumatic disease and severe pain in other body parts than the neck, hip, knee or ankle injuries, vestibular disorder, use of medication with side-effects on the postural control system	9; 44 (10); 6/3; 171 (10); 79 (14); NR	16; 41 (9); 13/3; 168 (8); 70 (14); NR	Age and sex	NR	Symptoms (pain) > 6 mon; 87 (77) mon	VAS (0–10) over the last week: 4.9 (2.3)
Pereira (2008) [31]	Whiplash injury due to motor vehicle accident > 3 mon No previously diagnosed vestibular dysfunction or associated diseases, positive Dix-Hallpike manoeuvre, previously diagnosed diseases of central nervous system, impaired visual acuity or known disorders of eye movement, deafness, hearing aids, ear surgery, vascular risk factors, migraine, known arteriosclerotic disease, history of dizziness or unsteadiness	30; 33.8 (9.4); 22/8; NR; NR; NR	30; 25.6 (5.1); 22/8; NR; NR; NR	Sex	NR	Injury > 3 mon	NDI (0–100): 60.2 (38.0) TSK: 38 (7.8)
Rushton (2014) [32]	Grade II WAD Symptom duration > 6 mon No history of neck pain or headache before injury, previous neuromusculoskeletal spinal presentations including surgery, osteoporosis or fracture, altered neurological sensory or motor function, other diseases known to induce peripheral neuropathy, diabetes, rheumatoid arthritis, epilepsy, HIV, tuberculosis, cancer, uncontrolled hypertension, current pregnancy or infection, peripheral nerve lesions, alcoholism, medications such as non-steroidal anti-inflammatory drugs	20; Median (IQR): 28.5 (12.8); 13/7; Median (IQR): 170.0 (11.3); Median (IQR): 73.5 (19.5); NR	22; Median (IQR): 26.0 (4.0); 9/13; Median (IQR): 174.0 (11.3); Median (IQR): 74.0 (18.3); NR	Age, sex, height and weight	20 Grade II	Symptoms > 6 mon Injury, Median (IQR): 46.5 (25.8) mon	NDI (0–100), Median (IQR): 21.0 (1.2)
Sjölander (2008) [33]	Grade II or III Neck pain duration > 6 mon No neurological disease, signs of brain damage, vestibular system impairment, rheumatic disease, severe pain in body regions	7; 45 (11); 5/2; 170 (10); 79 (13); NR	16; 41 (9); 13/3; 168 (8); 70 (14); NR	Age, height and weight	Grade II or III	Symptoms (pain) > 6 mon; 76 (84) mon	VAS (0–10), pain over the last week: 4.5 (1.9) NDI (0–100%): 44% (23%)
Sterling (2004) [34]	Grade II or III No Grade IV, concussion, loss of consciousness or head injury as a result of the accident, previous history of whiplash, neck pain, headaches, psychiatric condition that required treatment	80; 33.5 (14.7); 56/24; NR; NR; NR	20; 39.5 (14.6); 11/9; NR; NR; NR	Age and sex (adjusted)	77 Grade II; 3 Grade III	Injury < 1 mon	NDI (0–100%): 33.24%
Storaci (2006) [43]	Grade II No oculomotor dysfunction	40; 28.4 (8.8); 24/16; NR; NR; NR	40; 33.9 (12.7); 23/17; NR; NR; NR	NR	40 Grade II	Injury: 2.8 (4.3) mon	NR
Treleaven (2003) [35]	Grade II or III Whiplash injury > 3 mon No unconsciousness or concurrent head injury at the time of the accident, history of dizziness prior to the injury	WAD_D_ 76; Mean (SE): 39.11 (1.3); 54/22; NR; NR; NR WAD_ND_ 26; Mean (SE): 40.23 (1.9); 19/7; NR; NR; NR	44; Mean (SE): 34.1 (1.8); 29/15; NR; NR; NR	All groups: Age and sex	96 Grade II; 9 Grade III	Injury > 3 mon; WAD_D_, Mean (SE): 19.2 (5.6) mon; WAD_ND_, Mean (SE): 18.4 (6.0) mon	VAS (0–10), rest pain: Mean (SE) WAD D: 4.94 (0.25) WAD ND: 3.96 (0.40) NPQ (0–100%) WAD D: 55.3 (1.39) WAD ND: 43.1 (1.85)
Treleaven (2005) [11]	Grade II WAD Symptom duration > 3 mon post-injury with intermittent symptoms of dizziness and or unsteadiness at least once per week No unconsciousness or concurrent head injury with the whiplash injury, pre-existing diagnosed or suspected vestibular pathology, psychiatric conditions, neurological deficits and hip, knee or ankle pathology, use of medication that may adversely affect postural sway for 24 h prior to testing	20; 32.4 (19–45); 15/5; NR; NR; NR	20; 27.6 (19–45); 11/9; NR; NR; NR	NR	20 Grade II	Injury > 3 mon; 27.2 (4–60) mon	NR
Treleaven (2005) [44]	Grade II WAD Symptom duration > 3 mon post-injury WAD_D_: episodes of dizziness or unsteadiness at least twice per week, related to whiplash injury WAD_ND_: no dizziness or unsteadiness No unconsciousness, post-traumatic amnesia or concurrent head injury with the whiplash injury, known or suspected vestibular pathology such as benign paroxysmal positional vertigo, a history of dizziness prior to the whiplash injury, psychiatric conditions, neurological deficits and hip, knee or ankle pathology, medication such as anti-inflammatory, antipsychotic and narcotic medication for 24 hours prior to testing	WAD_D_ 50; Mean (SE): 35.6 (1.1); 38/12; NR; NR; NR WAD_ND_ 50; Mean (SE): 35.8 (1.3); 38/12; NR; NR; NR	50; 29.9 (1.4); 28/22; NR; NR; NR	All groups: Age and sex	100 Grade II	Symptoms > 3 mon; WAD _D_, Mean (SE): 1.4 (0.11) years; WAD _ND_, Mean (SE): 1.6 (0.14) years	VAS (0–10), rest pain: Mean (SE) WAD_D_: 4.1 (0.32) WAD_ND_: 2.8 (0.29) NDI (0–100%) WAD_D_: 46.4 (2.1) WAD_ND_: 34.4 (2.0)
Treleaven (2008) [36]	Whiplash injury due to motor vehicle collision Whiplash injury > 3 mon post-injury with dizziness and unsteadiness as a primary complain No unconsciousness, posttraumatic amnesia, concurrent head injury with the Whiplash injury, suspended vestibular pathology such as benign paroxysmal positional vertigo, a history of dizziness before the whiplash injury, psychiatric conditions, neurologic disorders, hip/knee/ankle pathology Able to turn the head to 45° right or left without increased pain,	20; 46.5 (40–60); 15/5; NR; NR; NR	20; 49.5 (43–59); 14/6; NR; NR; NR	NR	NR	Injury > 3 mon; 17 (4–36) mon	Dizziness Handicap Inventory- short form (0–13), Mean (SE): 7.6 (0.69)
Woodhouse (2008) [37]	Grade I or II WAD Motor vehicle accident, being either driver or passenger Symptom duration > 6 mon, < 10 years Onset of symptom within 48 hrs after accident No Grade III or IV WAD, head injury, history of surgery in the cervical spine, history of similar symptoms before accident, any systemic disease	59; 38.19 (10.8); 34/22	57; 38.2 (10.9); 28/29	Age and sex	Grade I or II	Symptoms > 6 mon, 10 years	NRS (0–10), current pain: 5.60 (2.49)
Yu (2011) [45]	Grade II WAD Age range: 18–50 years Whiplash injury from a motor vehicle collision Symptomatic > 3 mon post-injury Neck pain > 3 mon; NDI > 10/100 No current or past lower limb problems, known vestibular pathology, significant visual or hearing deficits, neurological deficits, Type II diabetes, abnormal blood pressure, diagnosed psychiatric disorders, loss of consciousness at the time of injury, a history of dizziness prior to the injury, taking medication such as antipsychotic and narcotic medication that may influence balance, consuming alcohol for at least 24 h prior to testing Excluded if they did not have at least 45 head rotation to the left and right	20; Mean (SE): 34.9 (1.8); NR; NR; NR; NR	20; Mean (SE): 30.25 (2.1); NR; NR; NR; NR	Age	20 Grade II	Symptoms > 3 mon Injury: 33.6 (10–60) mon	VAS (0–10), current rest pain, Mean (SE): 4.13 (0.6) NDI (0–100), Mean (SE): 46.5 (4.3) DHIsf (0–13), Mean (SE): 7.85 (0.6)

BMI: Body Mass Index; VAS: Visual Analogue Scale; NDI: Neck Disability Index; NRS: Numeric Rating Scale; NPQ: Northwick Park Neck Pain Questionnaire.

#### Description of outcome measures

Two types of target-matching tasks are commonly used to measure JPS; head repositioning to a neutral-head-position (HR-NHP) and head repositioning to a specific target remote from the NHP (HR-Target). In the HR-NHP task, the participant’s head is actively or passively brought from the ‘start position’ (a position far from NHP) to NHP, where NHP is the ‘target position’. While holding the head in the NHP for a short period, the participant is asked to memorize this target position. The head is then (actively or passively) moved away from the target position and the participant is asked to actively reposition the head back to the target position or to indicate the target position when the head is moved passively. In the HR-Target task, the same procedure as indicated for HR-NHP task is followed but the target position is now remote from NHP (such as 50° left rotation). The movements are performed in either horizontal (right and left rotation), sagittal (flexion, extension), or frontal (side-bending) plane. Repositioning error, as the primary outcome measure, is defined as the distance between the target position and the point indicated by the subject and is expressed as either absolute error (AE; average of absolute deviation from the target position, without regard to direction of error), constant error (CE; average deviation from the target position considering direction of error) or variable error (VE; SD of deviation from the target position) in either degrees or cm [29]. Errors are reported for either primary, secondary or combined primary and secondary (global) planes of movement.

HR-NHP task was reported in 15 [8, 10, 15, 25–29, 31–37] and HR-Target in 4 [8, 10, 15, 30] out of 16 included studies (Table 4). Fourteen studies measured HR-NHP by returning from rotation [10, 15, 25–29, 31–37], 12 studies from extension [9, 10, 25–29, 31, 32, 34–36] and 6 studies from flexion [10, 25–28, 32]. In 4 studies employing HR-Target task, the target was set at a specific point located in one of the primary planes, including rotation in all 4 studies [8, 10, 15, 30], extension in 1 study [10], flexion in 1 study [10] and lateral flexion in 1 study [30]. In all of the above-mentioned studies, the target position was replicated actively by the participants. All except 1 study expressed JPS performance as AE [8, 10, 15, 25–32, 34–37], 7 studies as CE [10, 26–29, 32, 33] and 4 studies as VE [10, 26, 29, 33]. Most studies reported JPS error for the primary plane of movement [8, 15, 25–29, 31–36]. Based on the number of times the above parameters were reported, we synthesized JPS AE in the primary plane of movement for separate movement directions.

**Table 4:** Measurement protocol of studies measuring joint position sense error.

First author (year)	Equipment/ instrument	Start position	Movement performed	Movement velocity	Target position/movement	Number of practice trials	Number of measurement trials	Outcome measure
Armstrong (2005) [10]	Electromagnetic tracking device (3-space Fastrak) Sensors placed on forehead, C3 and T1	Seated	Flexion, Extension, Left and Right Rotation	Self-selected pace	HR-NHP: Flexion, Extension, Right and Left Rotation (EC) HR-Target: Randomly selected mid-point within Flexion, Extension, Right and Left Rotation range (EC)	NR	3 per movement direction	AE, CE, VE (°) reported for pooled means of Flexion, Extension, Right and Left Rotation
De Pauw (2018) [25]	Laser pointer	Seated	Flexion, Extension, Left and Right Rotation	Self-selected pace	HR-NHP: Flexion, Extension, Right and Left Rotation	NR	10 per movement direction	AE (°) reported for primary, secondary and global plane of movement, averaged over Flexion and Extension and over Right and Left Rotation
Feipel (2006) [8]	Electrogoniometer (CA 6000 Spine Motion Analyzer) mounted on T1 and top of the head	Seated	Extension, Left and Right Rotation, Multiplanar movement	Self-selected pace	HR-NHP: Extension (EO and EC) HR-Target: 50° Left and Right Rotation (EC)	NR	Head to neutral-head position: 4 Head to 50° Rot: 3	AE (°), reported for primary and secondary plane of movement
Grip (2007) [26]	Optoelectronic (ProReflx System), markers placed on head and upper trunk	Seated	Flexion, Extension, Left and Right Rotation	Self-selected pace	HR-NHP: Flexion (25°), Extension (25°), Left and Right Rotation (30°) (EC)	NR	5 per movement direction	AE, CE, VE (°), reported for primary plane of movement
Heikkilä (1996) [27]	Laser pointer	Seated	Flexion, Extension, Left and Right Rotation	Self-selected pace	HR-NHP: Flexion, Extension, Left and Right Rotation (EC)	NR	10 per movement direction	CE (cm), reported for primary and secondary plane of movement AE (cm) reported for global plane of movement
Heikkilä (1998) [28]	Laser pointer	Seated	Flexion, Extension, Left and Right Rotation	Self-selected pace	HR-NHP: Flexion, Extension, Left and Right Rotation (EC)	NR	10 per movement direction	CE (cm), reported for primary and secondary plane of movement AE (cm) reported for global plane of movement
Hill (2009) [29]	Electromagnetic tracking device (3-space Fastrak) Sensors placed on forehead and C7	Seated	Extension, Left and Right Rotation	Self-selected pace	HR-NHP: Extension, Left and Right rotation (EC)	NR	3 per movement direction	AE, CE, VE (°), reported for primary plane of movement
Kristjansson (2003) [15]	Electromagnetic tracking device (3-space Fastrak) Sensors placed on forehead and C7	Seated	Rotation, Multiplanar movements	Self-selected pace	HR-NHP: Rotation (EC) HR-Target: 30° Right and Left Rotation (EC)	NR	3 per movement direction	AE (°), reported for primary plane of movement
Loudon (1997) [30]	Mechanical device, combining inclinometer + magnetic reference (Cervical Range of Motion device)	Seated	Right and Left Rotation, Right and Left Side-bending	Time limited: 60 sec	HR-Target: 30° Right Rotation, 30° Left Rotation, 50° Right Rotation, 50° Left Rotation, 20° Right Side-bending, 20° Left Side-bending (EC)	NR	3 per movement direction	AE (°)
Pereira (2008) [31]	Electromagnetic tracking device (3-space Fastrak)	Seated	Extension, Right and Left Rotation	Self-selected pace	HR-NHP: Extension, Right and Left Rotation (EC)	1	3	AE (°), reported for primary plane of movement
Rushton (2014) [32]	Laser pointer	NR	Flexion, Extension, Right and Left Rotation	Self-selected pace	HR-NHP: Flexion, Extension, Right and Left Rotation (EC)	1 per movement direction	6 per movement direction	CE (mm), reported for primary and secondary plane of movement AE (mm) reported for global plane of movement
Sjölander (2008) [33]	Electromagnetic tracking device (3-space Fastrak) Sensors placed on forehead and T1	Standing	Right and Left Rotation	As fast as possible	HR-NHP: Right and Left Rotation (EC)	NR	8 per movement direction	CE, VE (°), reported for primary plane of movement
Sterling (2004) [34]	Electromagnetic tracking device (3-space Fastrak)	Seated	Extension, Right and Left Rotation	Comfortable speed	HR-NHP: Extension, Right and Left Rotation (EC)	NR	3 per movement direction	AE (°), reported for primary plane of movement
Treleaven (2003) [35]	Electromagnetic tracking device (3-space Fastrak) Sensors placed on forehead and C7	Seated	Extension, Right and Left Rotation	NR	HR-NHP: Extension, Right and Left Rotation (EC)	1 per movement direction	3 per movement direction	AE (°), reported for primary and secondary plane of movement
Treleaven (2008) [36]	Electromagnetic tracking device (3-space Fastrak) Sensors placed on forehead and C7	Seated	Extension, Right and Left Rotation	NR	HR-NHP: Extension, Right and Left Rotation (EC)	NR	3 per movement direction	AE (°), reported for primary plane of movement
Woodhouse (2008) [37]	Electromagnetic tracking device (3-space Fastrak) Sensor place on the forehead	Seated	Right and Left Rotation	NR	HR-NHP: Right and Left Rotation (EC)	NR	2 per movement direction	AE (°), reported for the largest value of three planes, averaged over movement directions

HR-NHP: Head repositioning to neutral-head-position; HR-Target: Head repositioning to a specific target remote from neutral-head-position.

Feipel (2006) [8]: In contrast to other included studies that have used separate Flexion and Extension movement, the subjects in this study were requested to perform a full-range movement in the sagittal plane. They were requested to perform a maximal flexion, starting from NHP, followed by a maximal extension before returning to NHP. The movement before reproduction of NHP, i.e. extension component, was considered for meta-analysis in the present study.

Among various instruments used to measure JPS, including an electrogoniometer, laser pointer, optoelectronic and Cervical Range of Motion devices, 9 studies used electromagnetic devices to register head and neck movements. The number of repetitions per a specific target-matching task varied from 2 to 10, with 3 trials reported with greater frequency. With the exception of 1 study that measured HR-NHP in both EO and EC conditions [8], all others measured JPS in an EC condition.

#### Difference between people with WAD and HC

**Fig 2:**
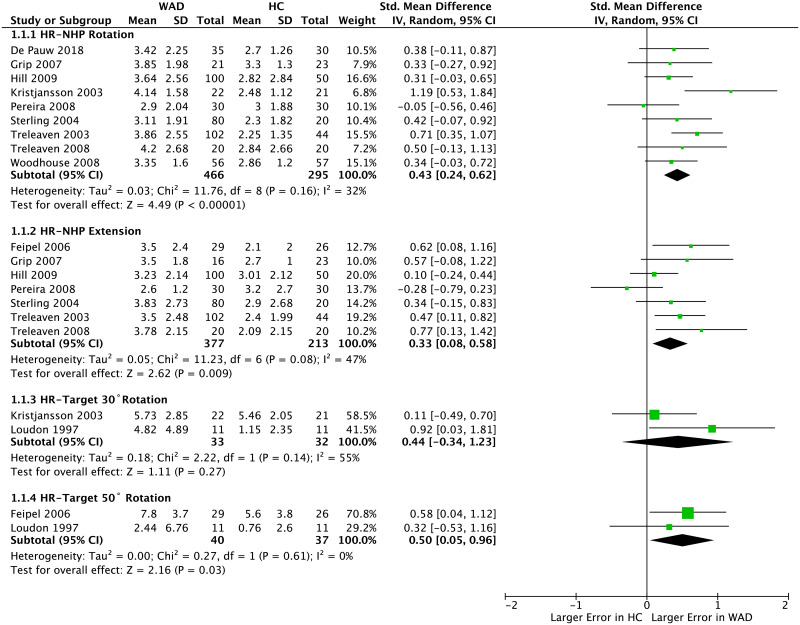
Forest plot demonstrating meta-analysis of absolute error (in degrees) of JPS when repositioning the head to neutral-head-position from extension and rotation and when repositioning the head toward targets set at 30° and 50° rotation from neutral-head-position in people with WAD vs. HC. WAD: whiplash-associated disorder; HC: healthy control; SD: standard deviation; IV: inverse variance; CI: confidence interval; Std: standardized.

Meta-analysis of 9 studies [15, 25, 26, 31, 34–37] that measured HR-NHP from rotation showed larger error in people with WAD compared to HC (pooled SMD = 0.43 [95%: 0.24–0.62], *p* < 0.001; *I*^2^ = 32%) (Fig 2). The results of 5 studies could not be synthesized as they averaged JPS error over different movements [10] or did not report AE for the primary plane of movement [27, 28, 32, 33].

Similar results were found when combining data of 7 studies [8, 26, 29, 31, 34–36] that measured AE of HR-NHP from extension, with larger error observed in people with WAD compared to HC (pooled SMD = 0.33 [95%CI: 0.08–0.58], *p* < 0.01; *I*^2^ = 47%) (Fig 2). Similar to rotation movements, data of 5 studies reporting HR-NHP from extension could not be pooled either because they averaged JPS error over different movements [10, 25] or did not report AE for the primary plane of movement [27, 28, 32].

**Fig 3:**
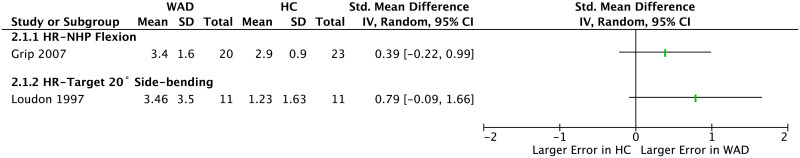
Forest plot, without meta-analysis, demonstrating absolute error (in degrees) of JPS when repositioning the head to neutral-head-position from flexion and when repositioning the head toward a target set at 20° side-bending from neutral-head-position in people with WAD vs. HC. WAD: whiplash-associated disorder; HC: healthy control; SD: standard deviation; IV: inverse variance; CI: confidence interval; Std: standardized.

Meta-analysis could not be performed for 6 studies which assessed HR-NHP from flexion due to either averaging JPS error over different movements [10, 25] or absence of AE for the primary plane of movement [27, 28, 32]. The only study [26] that provided AE of JPS from a pure flexion movement found no between-group difference (Fig 3).

For the HR-Target task, pooled results of 2 studies [15, 30] that provided data of repositioning towards 30° of rotation yielded no significant difference between the two groups (pooled SMD = 0.44 [-0.34–1.23], *p* = 0.27, *I*^2^ = 55%). However, those with WAD showed larger error compared to HC when data was pooled for 2 studies [8, 30] that assessed repositioning toward rotation at 50° (pooled SMD = 0.50 [0.05–0.96], *p*<0.05, *I*^2^ = 0) (Fig 2). One study that assessed JPS when repositioning toward rotation, flexion and extension [10] did not provide AE values for the primary plane of movement and hence was not included for a meta-analysis. No significant difference was found between the two groups in another study when the target was set at 20° side-bending [30] (Fig 3).

#### Difference between WAD_D_ and WAD_ND_ groups

**Fig 4:**
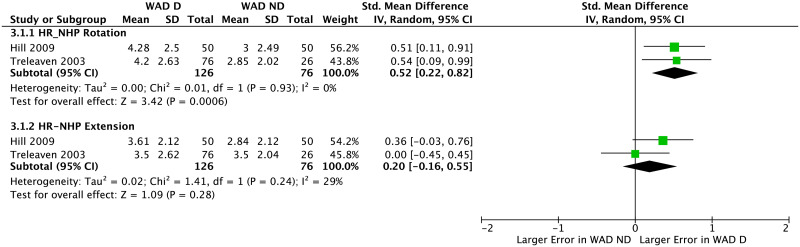
Forest plot demonstrating meta-analysis of absolute error (in degrees) of JPS when repositioning the head to neutral-head-position from extension and rotation in WAD_D_ group vs. WAD_ND_ group. WAD_D_: whiplash-associated disorder presenting with dizziness; WAD_ND_: whiplash-associated disorder not presenting with dizziness; SD: standard deviation; IV: inverse variance; CI: confidence interval; Std: standardized.

In both studies that assessed the difference between WAD_D_ and WAD_ND_ groups [29, 35], JPS was evaluated using HR-NHP from rotation and extension. For the HR-NHP task from rotation, larger error was observed for WAD_D_ group compared to WAD_ND_ group (pooled SMD = 0.52 [0.22–0.82], *p*<0.001, *I*^2^ = 0), whereas for the HR-NHP task from extension, no difference was observed between the two groups (pooled SMD = 0.20 [-0.16–0.55], *p* = 0.28, *I*^2^ = 29%) (Fig 4).

### Standing balance

#### Description of participants

For standing balance, the difference between people with WAD and HC was assessed in 12 studies [9, 11, 25, 36, 38–45], with 1 study investigating the difference between WAD_D_ and WAD_ND_ groups [45] (Table 3). These studies recruited a total of 698 participants, N = 390 participants with WAD (range: 9–100) and N = 308 HC (range: 10–50). One study [43] tested participants at 2.8 months post-injury (on average), while the other studies assessed standing balance in people with chronic WAD at least 3 months post-injury or initiation of symptoms. The average pain score, measured to indicate current pain in 3 studies [9, 41, 45] and pain over the last week in only 1 study [42] by either VAS [9, 42, 44, 45] or NRS [41], was 4.1 (range over studies: 3.2–4.9). Only 4 studies described the pain-related disability of their participants [9, 41, 44, 45], all utilizing the Neck Disability Index with an average score of 41.2%.

#### Description of outcome measures

In order to compare the contribution of sensory information into standing balance between people with WAD and HC, various sources of sensory feedback were manipulated including: (1) visual feedback by occluding the eyes in all 12 studies [9, 11, 25, 36, 38–45] or moving the visual surrounds in 3 studies [11, 36, 44], (2) proprioceptive feedback by alteration of support surface in 4 studies [9, 11, 36, 44], and (3) combination of visual and proprioceptive feedback by alteration of support surface along with either occluding the eyes in 4 studies [9, 11, 36, 44] or moving visual surrounds in 3 studies [11, 36, 44] (Table 5). This review analysed between-group differences in the two most common conditions of sensory manipulation, i.e., no-manipulation or EO and EC conditions.

**Table 5:** Measurement protocol of studies measuring standing balance.

First author (year)	Equipment	Test condition	Instruction	Foot and hand position	Duration of measurement trials	Number of measurement trials	Sampling frequency	Outcome measure
Bianco (2014) [38]	Electronic baropodometer	EO-FS EC-FS	To stand relax and to look at a fixed point	Parallel feet; arms at side	51.2 s	2 EO 2 EC	NR	Mean velocity AP velocity ML velocity Path length Area (Envelope) Path length/Area
De Pauw (2018) [25]	Force platform (AMTI)	EC-FS	NR	Feet placed at hip width	90 s	3	100 Hz	Mean velocity Area (95% confidence ellipse)
Endo (2008) [39]	Force platform (Anima)	EO-FS EC-FS	To look at a fixed point	NR	60 s	1 per condition	NR	Area (Envelop) in unit of time Path length/ second
Eriksson (2019) [40]	Video-based camera system, markers placed on the head	EO-FS EC-FS	To stand upright	Feet together	120 s	4 EC 3 EO	50 Hz	Area (perimeter of sway movement area) Speed Acceleration
Field (2008) [9]	Force platform	**Comfortable stance**: EO-FS EC-FS EO-SS EC-SS **Narrow stance**: EO-FS EC-FS EO-SS EC-SS	To stand as steadily as possible and to look at a fixed point	Feet placed at preferred width in comfortable stance condition and feet together in narrow stance condition; arms at side	30 s	1 per condition	15 Hz	Amplitude (RMS) in AP and ML direction
Juul-Kristensen (2013) [41]	Force platform (AMTI)	EO-FS EC-FS	To look at a fixed point	Feet together	30 s	1 EO 3 EC	125 Hz	Area (95% confidence ellipse) Range AP Range ML
Michaelson (2003) [42]	Force platform (Kistler)	EO-FS EC-FS	To stand as quite as possible and to look at a fixed point	Feet together; arms crossed over the chest	30 s	-	30 Hz	Area (95% confidence ellipse)
Storaci (2006) [43]	Static posturography platform	EO-FS EC-FS	NR	NR	51.2	2 EO 1 EC	NR	Area (90% confidence ellipse) Path length Path length/area
Treleaven (2005) [11]	Force platform	EO-FS EO-SS EC-FS EC-SS Visual conflict-FS Visual conflict-SS	To look at a fixed point	Feet placed in comfortable position; arms at side	30 s	1	NR	Path length
Treleaven (2005) [44]	Force platform	EO-FS EO-SS EC-FS EC-SS EC-SS Visual conflict-FS Visual conflict-SS	To stand as quite as possible and to look at a fixed point	Feet placed in comfortable position; arms at side	30 s	1 per condition	NR	Sway energy
Treleaven (2008) [36]	Force platform	**Comfortable stance**: EO-FS EO-SS EC-FS EC-SS EC-SS Visual conflict-FS Visual conflict-SS **Narrow stance**: EO-FS EO-SS EC-FS EC-SS EC-SS	NR	Feet placed in comfortable position and feet together in narrow stance condition	30 s	1 per condition	NR	Sway energy

Traditional time-domain COP measures used in the included studies included sway area, perimeter of sway area, velocity (mean, anteroposterior [AP] and mediolateral [ML]), path length, path length per second, path length per area, acceleration, amplitude and range. The traditional time-domain COP parameters were categorized into sway velocity (velocity, path length and path length per second) and sway amplitude (sway area, perimeter of sway area, amplitude and range) [46]. We did not include path length per area and acceleration in either amplitude or velocity categories. If more than one COP parameter was reported in a single study, only one parameter representing COP velocity or amplitude was selected for meta-analysis and changes in other parameters were described narratively. This selection was based on prioritizing total sway over sway in specific directions as well as an arbitrary selection of a COP parameter when several parameters provide the same information, such as path length, path length per second and velocity. Two studies [36, 44] only used frequency-domain COP measures to quantify standing balance. The measure used in these studies was sway energy, i.e. the amount of energy of the power spectrum of the COP signal.

A force platform was most commonly used to measure standing balance [9, 11, 25, 36, 39, 41, 42, 44, 45], whereas an electronic baropodometer [38] was used in one study and a video-based camera system [40] in another. The instrument in one study was not clearly described [43]. Foot placement during quiet standing on the force platform differed, ranging from feet together [9, 36, 40–42] to feet apart (comfortable) [9, 11, 25, 36, 44, 45]. Few studies provided information regarding the instruction given explicitly to the participants and it varied from ‘to stand as still as possible’ [9, 42, 44, 45] to ‘stand relaxed’ [38]. In most studies [9, 11, 38, 39, 41, 42, 44, 45], participants were asked to look at a fixed point during standing. The duration of postural assessment ranged from 30 s to 120 s, with 30 s reported more commonly. The number of measurement trials ranged from 1 to 4 trials, but less than 3 trials was used more commonly.

#### Difference between people with WAD and HC

**Fig 5:**
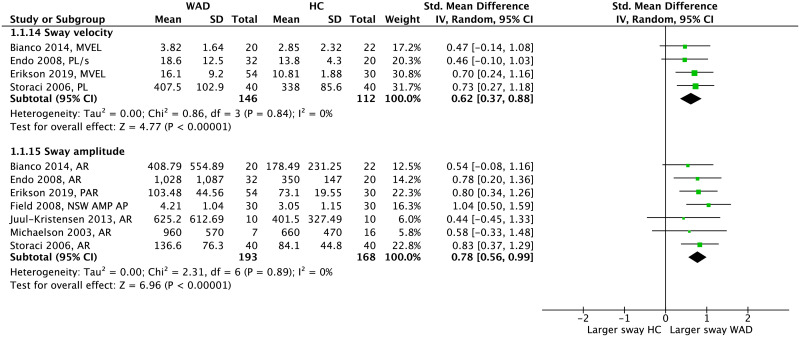
Forest plot demonstrating meta-analysis of sway velocity and sway amplitude (eyes open) in people with WAD vs. HC. WAD: whiplash-associated disorder; HC: healthy control; SD: standard deviation; IV: inverse variance; CI: confidence interval; Std: standardized; MVEL: mean velocity (mm/s); PL: path length (mm); AR: area (mm^2^); PAR: perimeter of sway area (mm); AMP: amplitude (mm); AP: anteroposterior; NSW: narrow stance width.

**Fig 6:**
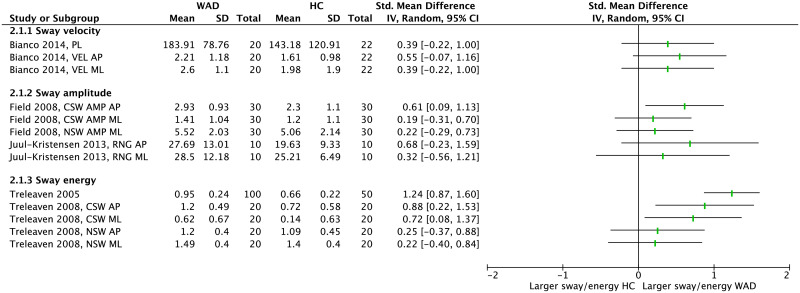
Forest plot, without meta-analysis, demonstrating sway velocity, sway amplitude and sway energy (eyes open) in people with WAD vs. HC. WAD: whiplash-associated disorder; HC: healthy control; SD: standard deviation; IV: inverse variance; CI: confidence interval; Std: standardized; VEL: velocity (mm/s); PL: path length (mm); AMP: amplitude (mm); RNG: range (mm); AP: anteroposterior; ML: mediolateral; CSW: comfortable stance width; NSW: narrow stance width.

Meta-analysis of COP measures in the EO condition showed that people with WAD demonstrate significantly larger postural sway in terms of velocity (pooled SMD = 0.62 [0.37–0.88], *p*<0.001, *I*^2^ = 0) and amplitude (pooled SMD = 0.78 [0.56–0.99], *p*<0.001, *I*^2^ = 0) compared to HC (Fig 5). From time-domain parameters that were not included in a meta-analysis all except one showed no between-group difference (Fig 6). Among the frequency-domain measures, 3 out of 5 parameters showed larger sway energy in people with WAD compared to HC.

**Fig 7:**
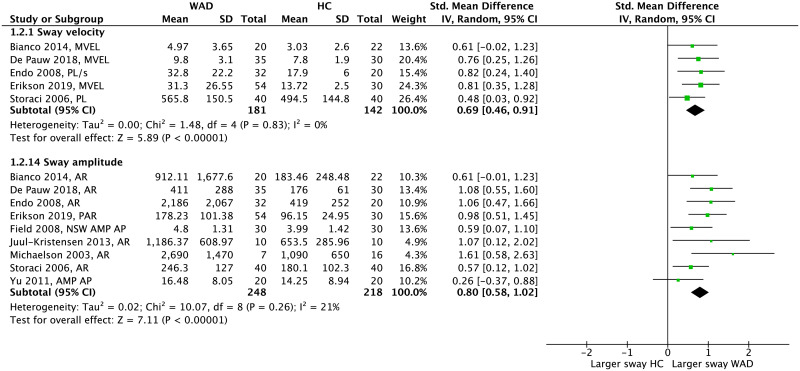
Forest plot demonstrating meta-analysis of sway velocity and sway amplitude (eyes closed) in people with WAD vs. HC. WAD: whiplash-associated disorder; HC: healthy control; SD: standard deviation; IV: inverse variance; CI: confidence interval; Std: standardized; MVEL: mean velocity (mm/s); PL: path length (mm); AR: area (mm^2^); PAR: perimeter of sway area (mm); AMP: amplitude (mm); AP: anteroposterior; NSW: narrow stance width.

**Fig 8:**
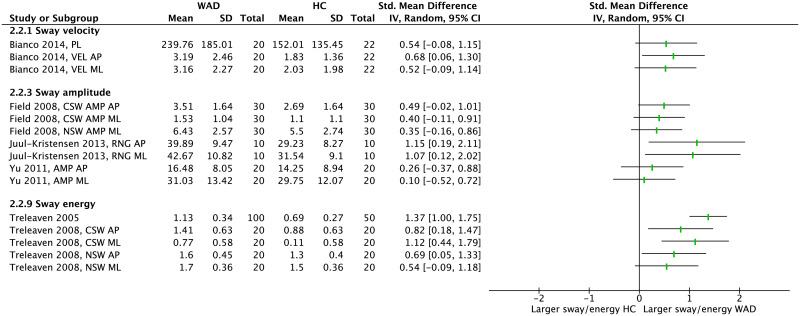
Forest plot, without meta-analysis, demonstrating sway velocity, sway amplitude and sway energy (eyes closed) in people with WAD vs. HC. WAD: whiplash-associated disorder; HC: healthy control; SD: standard deviation; IV: inverse variance; CI: confidence interval; Std: standardized; VEL: mean velocity (mm/s); PL: path length (mm); RNG: range (mm); AMP: amplitude (mm); AP: anteroposterior; ML: mediolateral; CSW: comfortable stance width; NSW: narrow stance width.

Synthesis of COP parameters in the EC condition showed a significant effect for both sway velocity (pooled SMD = 0.69 [0.46–0.91], *p*<0.001, *I*^2^ = 0) and amplitude (pooled SMD = 0.80 [0.58–1.02], *p*<0.001, *I*^2^ = 21%) indicating that people with WAD show larger sway compared to HC (Fig 7). Among time-domain parameters that were not present in the meta-analysis, only 3 out of 10 showed significant findings that were consistent with the overall effect obtained from meta-analysis (Fig 8). Within the frequency-domain measures, 4 out of 5 parameters showed larger sway energy in people with WAD compared to HC.

#### Difference between WAD_D_ and WAD_ND_ groups

**Fig 9:**
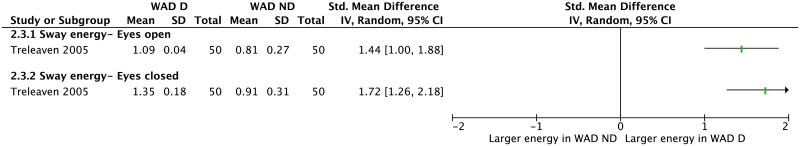
Forest plot, without meta-analysis, demonstrating sway energy (eyes closed) in WAD_D_ vs. WAD_ND_ groups. WAD_D_: whiplash-associated disorder presenting with dizziness; WAD_ND_: whiplash-associated disorder not presenting with dizziness; SD: standard deviation; IV: inverse variance; CI: confidence interval; Std: standardized.

In a single study that compared WAD_D_ and WAD_ND_ groups, larger sway energy was observed in WAD_D_ compared to WAD_ND_ in both EO and EC conditions (Fig 9).

### Methodological quality

Most of the included studies provided information regarding the aim or hypothesis (JPS: 16/16 and standing balance: 12/12), inclusion and exclusion criteria (13/16 and 10/12) and background characteristics (16/16 and 11/12) of the participants (Table 6).

**Table 6:** Quality rating of included studies using the modified Downs and Black Scale.

Studies	Reporting						External validity	Internal validity			Power	Score (out of 11)
	1	2	3	4	5	6	7	8	9	10	11	
**Joint position sense**												
Armstrong (2005) [10]	✔	✔	✔	✔	✔	✔	✘	✘	✘	✔	✘	7
De Pauw (2018) [25]	✔	✔	✔	✔	✘	✔	✘	✘	✔	✔	✔	8
Feipel (2006) [8]	✔	✔	✔	✘	✘	✔	✘	✘	✘	✔	✘	5
Grip (2007) [26]	✔	✘	✔	✔	✘	✔	✘	✘	✘	✘	✘	4
Heikkila (1996) [27]	✔	✘	✔	✘	✘	✔	✘	✘	✔	✘	✘	4
Heikkila (1998) [28]	✔	✔	✔	✘	✘	✔	✘	✘	✔	✘	✘	5
Hill (2009) [29]	✔	✔	✔	✘	✘	✔	✔	✘	✘	✘	✘	5
Kristjansson (2003) [15]	✔	✔	✔	✘	✘	✔	✔	✔	✘	✔	✘	7
Loudon (1997) [30]	✔	✘	✔	✘	✔	✔	✘	✘	✘	✔	✘	5
Pereira (2008) [31]	✔	✔	✔	✘	✘	✔	✔	✘	✘	✘	✘	5
Rushton (2014) [32]	✔	✔	✔	✔	✘	✔	✘	✔	✔	✔	✘	8
Sjölander (2008) [33]	✔	✔	✔	✔	✘	✔	✘	✔	✔	✘	✘	7
Sterling (2004) [34]	✔	✔	✔	✔	✘	✔	✔	✘	✘	✔	✘	7
Treleaven (2003) [35]	✔	✔	✔	✔	✘	✔	✘	✘	✘	✔	✘	6
Treleaven (2008) [36]	✔	✔	✔	✘	✘	✘	✘	✘	✘	✘	✔	4
Woodhouse (2008) [37]	✔	✔	✔	✔	✘	✔	✘	✔	✘	✔	✘	7
**Standing balance**												
Bianco (2014) [38]	✔	✔	✔	✘	✘	✔	✘	✘	✘	✔	✘	5
De Pauw (2018) [25]	✔	✔	✔	✔	✘	✔	✘	✘	✔	✔	✔	8
Endo (2008) [39]	✔	✔	✔	✘	✘	✔	✘	✘	✘	✘	✘	4
Eriksson (2019) [40]	✔	✘	✔	✘	✔	✔	✘	✘	✔	✘	✘	5
Field (2008) [9]	✔	✔	✔	✔	✘	✘	✘	✘	✘	✔	✘	5
Juul-Kristensen (2013) [41]	✔	✔	✔	✘	✔	✔	✘	✔	✔	✔	✔	9
Michaelson (2003) [42]	✔	✔	✔	✘	✔	✔	✘	✔	✘	✔	✔	8
Storaci (2006) [43]	✔	✘	✔	✘	✘	✔	✘	✘	✘	✘	✘	3
Treleaven (2005) [11]	✔	✔	✔	✘	✘	✘	✘	✘	✘	✘	✘	3
Treleaven (2005) [44]	✔	✔	✔	✔	✘	✘	✔	✘	✘	✔	✘	6
Treleaven (2008) [36]	✔	✔	✔	✘	✘	✘	✘	✘	✘	✘	✔	4
Yu (2011) [45]	✔	✔	✘	✔	✘	✔	✘	✘	✘	✘	✘	4

For JPS and standing balance, clinical characteristics of people with WAD (injury grade and time since injury or initiation of symptoms and pain or disability level) was reported in a half and a third of studies, respectively. Information regarding treatment history was rarely reported (2/16 and 3/12). With a few exceptions where quantitative values were presented in figures, the remaining studies (15/16 and 8/12) detailed estimates of random variability, i.e. mean and SD or SEM.

Only one of the included studies investigating standing balance and very few (4/16) examining JPS recruited participants using multiple recruitment methods such as self-report, hospital or insurance companies- a factor that limits the generalizability of the findings.

Few studies in both groups of studies blinded the assessors of outcome measures to the participants (4/16 and 2/12). For JPS, less than a third of the included studies (5/16) used at least 6 trials to calculate JPS error. In studies of balance, 3/12 studies reported recording of COP at least 3 times and/or more than 90 sec (per measurement trial), whilst poor reliability was obtained for other studies. People with WAD and HC were comparable (matched or statistically adjusted) in terms of age and sex in approximately half of the studies measuring JPS (9/16) and standing balance (6/12). Sample size was justified in a very limited number of studies (2/16; 4/12).

The overall quality was similar between 5 studies examining JPS [8, 15, 30, 35, 36] that found at least one significant difference between people with WAD and HC and 6 studies [25, 26, 29, 31, 34, 37] that did not find any difference between the two groups (Mann-Whitney *U* = 11.5; *p* = 0.51). Inspection of individual methodological quality items showed that studies finding an effect obtained lower scores for item #4 (description of clinical characteristics; 1/5 studies reporting a difference vs. 4/6 studies reporting no difference) and item #7 (use of several recruitment methods; 1/5 vs. 3/6 respectively), but higher scores were noted for item #10 (matching groups; 4/5 vs. 3/6). Only one study among the studies which found a difference between WAD and HC used a reliable measure JPS whereas none of studies among those which found no difference used a reliable JPS measure. Even though a similar proportion of studies in both groups described or justified power or sample size, the median sample size of the studies finding no difference (n = 83) was nearly twice that of the studies that did report a difference (n = 43). Specifically, 3 studies finding no difference tested at least 100 participants [29, 34, 37], whilst only one study [35] which did report a difference, utilized sample of 100 participants. Due to poor reporting of subject clinical characteristics, we could not verify the role of factors such as WAD grade, pain/symptom duration/intensity or disability level in explaining differences between studies. All studies testing standing balance apart from 1 [45], found at least one significant difference in either the EO or EC conditions between people with WAD and HC, making the comparison of methodological quality meaningless.

## Discussion

This review assessed whether JPS and standing balance are altered in people with WAD compared to asymptomatic individuals and whether the extent of change is greater in those presenting with the symptom of dizziness. Results of the meta-analysis showed moderately larger JPS error in people with WAD compared to HC when the head was repositioned to a NHP from extension and rotation or when the head was moved toward 50° rotation from a NHP. Similarly, WAD_D_ group performed markedly worse compared to WAD_ND_ group when the head was repositioned to a NHP from rotation. Meta-analysis of standing balance studies showed markedly larger and faster sway in people with WAD compared to HC during conditions with the EO or EC. In a single study that took the presence or absence of dizziness into account [44], WAD_D_ group showed larger sway energy compared to WAD_ND_ group.

### Joint position sense—Difference between people with WAD and HC

People with WAD performed poorer compared to HC when the head was repositioned to a NHP starting from either rotation or extension. Although repositioning to a NHP from flexion was not significant between groups, this could be due to lack of power as this finding is based on a single study [26] with a relatively small sample size.

For target-matching tasks locating the target far from the NHP, people with WAD produced larger errors compared to HC but only when the head was repositioned to 50° of rotation. This result is in contrast to the results of Kritjansson et al. [15] who found that HR-Target is not as good as HR-NHP to differentiate people with WAD and HC, but the limited number of studies investigating HR-Target tasks makes it hard to draw firm conclusions regarding the discriminative ability of various target matching tasks. The results showed no difference between groups when the head was repositioned to 30°. Assuming that positions farther from the NHP are less practiced or learned and rely more on proprioceptive input, repositioning the head to 50° of rotation could better differentiate people with WAD versus HC compared to rotation at 30°. However, this proposition has no support since the difference between groups was evident when the head is repositioned to the most learned position, i.e. NHP. Taking into account the similar magnitude of SMD between 30° and 50°, we would suggest that these differing results are likely due to underpowered studies examining the 30° of rotation target-matching task. Further studies are needed to examine positioning to different points in range.

Even though the overall quality was similar between studies that did find a difference compared to those that did not, a detailed inspection of other possible explanatory factors showed that the sample size was surprisingly larger in the studies showing no difference compared to the studies demonstrating a difference. This may indicate either stricter inclusion criteria leading to more homogenous groups or stricter experimental control resulting in higher sensitivity in the smaller studies. Recruitment of participants with WAD in specific settings as well as tight control of the WAD and HC groups for age and sex in the studies which did report group differences supports homogeneity of the studied population. Finding a between group difference can also be attributed to the use of more reliable measures of JPS, as suggested by de Vries et al. [12]. In their review, de Vries et al. found that all studies finding a difference between people with neck pain and HC, calculated JPS error over at least 6 trials. In contrast, we found no difference in reliability of JPS error between studies that found an effect and those that did not find an effect. This discrepancy could be attributed to the fact that de Vries et al. combined all the groups (traumatic and non-traumatic neck pain) and outcome measures (AE and CE, movement directions) together.

### Joint position sense—Difference between WAD_D_ and WAD_ND_ groups

*WAD*_*ND*_ group performed worse compared to *WAD*_*ND*_ group only when the head was repositioned to a NHP from rotation but not from extension. One plausible explanation is that the rotation movement is accompanied by activation of the vestibular system in addition to proprioceptive system, in contrast to extension movement where there is less stimulation of the vestibular system [35]. Therefore, in rotation, the mismatch between sensory organs, i.e. abnormal proprioceptive and normal vestibular input, may be more pronounced, resulting in larger JPS error in people complaining of dizziness.

### Standing balance—Difference between people with WAD and HC

The results of the meta-analysis showed larger and faster sway in both visual conditions, i.e. EO and EC, in people with WAD compared to HC. As additional sensory input provided by visual feedback is supposed to compensate for impaired proprioceptive feedback in conditions with the EO, observation of similar results between EO and EC came as a surprise. For instance, in contrast to WAD, sensorimotor deficits due to anterior cruciate ligament injury [46] are detected in EC, but not EO conditions. Independence of postural sway to visual feedback in people with WAD may highlight the significant impact of impairment of either cervical afferent input, vestibular input or combination of both on standing balance even when other sources of sensory information are still available.

Absence of a significant effect of WAD on COP parameters not included in the meta-analysis could be attributed to lack of statistical power.

### Standing balance—Difference between WAD_D_ and WAD_ND_ groups

WAD_D_ group showed poorer standing balance compared to WAD_ND_ group. This was evident as larger sway energy in those people who complained of dizziness. This indicates higher effort needed by the postural control system to overcome the instability induced by postural fluctuations when dizziness is present.

### Sensorimotor control deficits in people with WAD

Damage to any element involved in sensorimotor control can lead to deficits of JPS or standing balance. Nociceptive inputs can induce changes at multiple levels of sensorimotor system including spinal and supra-spinal levels, which may contribute to disturbed sensorimotor deficits [47, 48]. Persistence of proprioceptive deficits may occur even after the resolution of acute pain [49]. The contribution of vestibular dysfunction in sensorimotor deficits cannot be ruled out, but the differing results of standing balance between people with WAD and non- traumatic vestibular pathology supports a cervical origin of somatosensory deficits in WAD [36]. People with WAD show larger deficits across the majority of sensory- and surface-type manipulations when compared to people with vestibular disorders and HC [36]. Therefore, the results of the both JPS and standing balance assessments highlight a likely modification of cervical afferent input in people with WAD.

Our findings confirm a greater disturbance of sensorimotor control in people with WAD presenting with dizziness compared to those that do not have dizziness. If dizziness in the WAD_D_ group is mainly attributed to a vestibular disorder, we would expect impaired balance but not necessarily JPS in WAD_D_ compared to WAD_ND_. Nevertheless, we cannot exclude the presence of vestibular dysfunction, in least in some participants. Further support for a disturbance in cervical afferent input, rather than vestibular, as a cause of sensorimotor deficits in people with WAD originates from findings of altered JPS and standing balance in people with no dizziness, as demonstrated by larger JPS error [29, 35] or larger sway energy [44] in those with WADND compared to HC. The symptom of dizziness might be due to, or related to, more severe disturbance of proprioceptive inputs in WAD_D_ group compared to WAD_ND_ group. Poor performance in other domains of sensorimotor control, such as oculomotor control [50], in people with WAD with the symptom of dizziness compared to those without dizziness, further supports the role of altered cervical somatosensory input in developing the symptom of dizziness.

Since some the above-mentioned mechanisms, such as alteration of proprioceptive feedback resulting from pain and/or injury, may have consequences for both JPS and standing balance, we may expect a high correlation between these two measures. However, as demonstrated by Treleaven et al. [51] in people with WAD, a strong linear association between JPS and standing balance does not exist. In other words, poor performance in one domain does not imply poor performance in the other domain. This can be attributed to different neural networks underlying JPS and postural control. For instance, cerebellum activation has been consistently reported in human and animal neuroimaging studies of static postural control [52]. However, findings of neural correlates of proprioception does not support a clear involvement of the cerebellum [53]. Although WAD related impairments may affect both domains, the nature of impairments may differ across people with WAD, and therefore the effects of WAD on JPS and standing balance may differ across individuals. These observations and deductions may suggest that JPS and standing balance are two separate constructs implying the need to assess both domains in clinical practice of people with WAD. Nevertheless, we do not exclude the existence of a relationship, as the moderate effect sizes of both measures JPS and balance measures may hinder detection of such an association in a sample of limited size.

The results of the current review highlight the existence of deficits in JPS and standing balance in people with WAD, especially in those with dizziness. These findings imply the importance of incorporation of assessment and management of these specific domains of sensorimotor control within the clinical management of people with WAD. The interventions should address the causes of altered cervical afferent input, such as pain and inflammation, morphological muscle changes or psychological stress, as well as secondary adaptive changes in the sensorimotor control system, such as altered coordination between cervical, visual and vestibular systems [54].

### Limitations

Various tests have been developed to measure JPS, but our review included only studies utilizing a specific test, i.e. target matching tasks by moving the head on a stationary trunk. Due to the uniqueness of different tests, the results of the current review are only applicable to target matching task. However, this is of minor concern as the majority of studies investigating JPS in people with WAD have employed HR-NHP and HR-Target tasks. Furthermore, these tasks have shown higher ability to discriminate between HC and people with WAD compared to other complex JPS tests [15]. We analysed AE as the measure of JPS since it was more frequently reported in the included studies. Preferential analysis of AE may limit extrapolation of study findings to other types of error, i.e. CE and VE. Furthermore, due to heterogeneity of outcomes measures in both JPS and standing stability domains, we analysed some studies using a narrative method which is a less rigorous method compared to meta-analysis. A very limited number of studies addressed the effect of dizziness associated with WAD on JPS and standing balance, which should be considered when interpreting the conclusions.

Few studies reported treatment history in the WAD group which could bias the results because they may have affected standing balance and JPS. Other quality criteria which were not adequately addressed involved describing clinical characteristics, blindness of assessors, reliability of measures, matching the comparison groups and power justification.

### Direction for future research

To date, very few studies have measured sensorimotor deficits in the acute phase. With the exception of two studies, one assessing JPS within 1 month post-injury [34] and the other examining standing balance 2.8 months post-injury (on average) [43], all studies in both groups included participants with chronic WAD. Even though the results of these studies indicate disturbance of JPS and standing balance, future studies should be conducted to determine how quickly these deficits occur following the onset of pain/dizziness and to what extent these disturbances tend to increase or decrease over time.

## Conclusions

This review found impairment of JPS and standing balance in people with WAD compared to HC. Altered JPS was more pronounced in WAD_D_ group compared to WAD_ND_ group. Even though similar underlying mechanisms are proposed to cause impaired JPS and standing balance, further research is needed to clarify if and how these impairments are related.

## Supporting information

S1 Checklist PRISMA 2009 checklist. (DOC)
